# The value of renal score in both determining surgical strategies and predicting complications for renal cell carcinoma: A systematic review and meta‐analysis

**DOI:** 10.1002/cam4.2993

**Published:** 2020-04-12

**Authors:** Naipeng Shi, Feng Zu, Yong Shan, Shuqiu Chen, Bin Xu, Mulong Du, Ming Chen

**Affiliations:** ^1^ Department of Urology Affiliated Zhongda Hospital of Southeast University Nanjing China; ^2^ Department of Urology Funing People's Hospital Yancheng Jiangsu China; ^3^ Department of Urology The Second People's Hospital of Taizhou Taizhou Jiangsu China; ^4^ Department of Environmental Genomics Jiangsu Key Laboratory of Cancer Biomarkers, Prevention and Treatment Collaborative Innovation Center for Cancer Personalized Medicine Nanjing Medical University Nanjing China

**Keywords:** complications, meta‐analysis, Renal cell carcinoma, RENAL score, surgical strategies

## Abstract

**Objectives:**

Radical nephrectomy (RN) was the standard treatment for renal cell carcinoma (RCC). However, recent studies have found that partial nephrectomy (PN) could achieve similar effects as radical nephrectomy, and has the advantages of less bleeding and shorter hospital stay. The choice of surgical strategies has become a concern of clinicians, which could be guided by renal score introduced by Kutikov et al Therefore, we conducted this meta‐analysis to clarify the value of renal score of determining surgical strategies and predicting complications.

**Methods:**

The keywords “RENAL score,” “renal nephrometry score,” or “nephrometry score” were used to retrieve electronic databases for relevant literature up to Feb 2020, including PubMed, Web of Science, and the Cochrane library. Surgical strategies and complications are outcome measures. Risk ratio (RR) with 95% confidence intervals (CI) is applied to assess the effect size.

**Results:**

A total of 20 studies met the selection criteria for meta‐analysis. There was significant difference in RN operation rate for each subgroup (low‐moderate: RR = 3.50, 95% Cl = 2.60‐4.71, *P* < .001; low‐high: RR = 6.29, 95% Cl = 4.40‐9.00, *P* < .001; moderate‐high: RR = 1.80, 95% Cl = 1.39‐2.32, *P* < .001).The overall incidence of complications from high renal score group was significantly higher than that in low renal score group (low‐moderate: RR = 1.32, 95% Cl = 1.03‐1.69, *P* = .026; low‐high: RR = 2.45, 95% Cl = 1.48‐4.07, *P* = .001; moderate‐high: RR = 1.75, 95% Cl = 1.17‐2.61, *P* = .007).

**Conclusions:**

This meta‐analysis indicated that renal score is an efficient tool for determining surgical strategies and predicting complications in PN. More prospective research is essential to verify the predictive value of renal score.

## Graphical abstract

The predictive value of renal score is unclear. We retrieved all studies on renal score and performed a meta‐analysis of surgical strategies and postoperative complications. The results show that renal score is an objective and effective tool for predicting surgical strategies and complications after PN. 

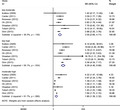

## 1 INTRODUCTION

Globally, renal cell carcinoma (RCC) is the sixth most commonly diagnosed cancer in men and the tenth most commonly diagnosed cancer in women, accounting for 5% and 3% of all tumors respectively.^1^ This has been fueled by an increase in incidentally diagnosed tumors on radiological imaging such as ultrasonography and computerized tomography (CT).^2^ In the past, radical nephrectomy (RN) was considered the standard treatment for renal cell carcinoma.^3^ However, the application rate of partial nephrectomy (PN) has been increasing in recent years, which may be related to the advantages of higher retention rate of renal function, shorter hospitalization time and less intraoperative bleeding. In addition, some studies have shown that PN is superior to RN in overall survival, cancer‐specific survival, and recurrence‐free survival.^4^, ^5^ It is also reported to be demanding and technically challenging for complex renal tumors.^6^ In order to select appropriate surgical strategies and better predict complications, several renal cancer scoring systems for describing the relevant renal mass anatomy have emerged as the times require.

The RENAL Nephrometry Score is a reproducible standardized classification system that quantitates the salient anatomy of renal masses.^7^ Several studies have been published on the relationship between renal score and surgical strategies and complications. However, due to the small scale of these studies, their results are various, which cannot accurately reflect the value of renal score. This systematic review and meta‐analysis is aimed at integrating all relevant studies on the relationship between renal scores and surgical strategies and complications and summarizing their results to accurately reflect the value of renal score in determining surgical strategies and predicting complications.

## 2 MATERIALS AND METHODS

This study was performed according to the Preferred Reporting Items for Systematic Reviews and Meta‐Analyses (PRISMA) statement.

### 2.1 Data resources and search strategies

A comprehensive search for relevant studies on the relationship between renal score and surgical strategies and complications until Feb 2020 was performed in appropriate electronic databases and sources, including PubMed, Web of Science, and the Cochrane library. The language was restricted to English. The following keywords or free terms were used: "RENAL score," "renal nephrometry score," or "nephrometry score." The reference lists of included studies were also manually reviewed to avoid missing relevant studies.

### 2.2 Inclusion and exclusion criteria

Inclusion criteria of the study are as follows: (a) Prospective or retrospective studies; (b) Patients underwent nephrectomy; (c) Patients were divided into three cohorts according to renal score as low (sum 4‐6), moderate (sum 7‐9), and high (sum 10‐12); (d) the number of events of interest outcome can be obtained.

Exclusion criteria: (a) Repeated reports; (b) Defects in research design and poor quality; (c) Incomplete or unavailable data and unclear outcomes.

### 2.3 Data extraction and study quality assessment

All data were extracted by two independent reviewers and the discrepancies were resolved by discussion with a third expert adjudicator. The following data were extracted from the literature: authors, year of publication, study design, and number of patients, etc The methodological quality of all of the included studies was independently assessed in duplicate by two reviewers with Newcastle‐Ottawa Quality Assessment Scale. A consensus between the two reviewers was reached for individual category scores.

### 2.4 Statistical analysis

We performed a meta‐analysis of the comparisons of RN operation rates and complication rates among three cohorts. Three groups of cohorts were compared in pairs, including low‐moderate, low‐high, and moderate‐high, in which relatively low renal score group served as a control group and the other one as an experimental group. All data were analyzed using Stata 14.0 Soft. Pooled risk ratio (RR) with 95% CIs was calculated to assess the effect size of renal score complexity. Heterogeneity among studies was analyzed using the *χ* ^2^ and I^2^ tests. The random effect model was used to calculate the pooled effect when I^2^ was > 50%. Otherwise, a fixed‐effects model was used. Meta regression analysis was performed to look for possible sources of heterogeneity. *P* < .05 was considered to indicate statistical significance. The publication bias was assessed for each comparison pair using Egger Test.

## 3 RESULTS

### 3.1 Data retrieval

**Figure 1:**
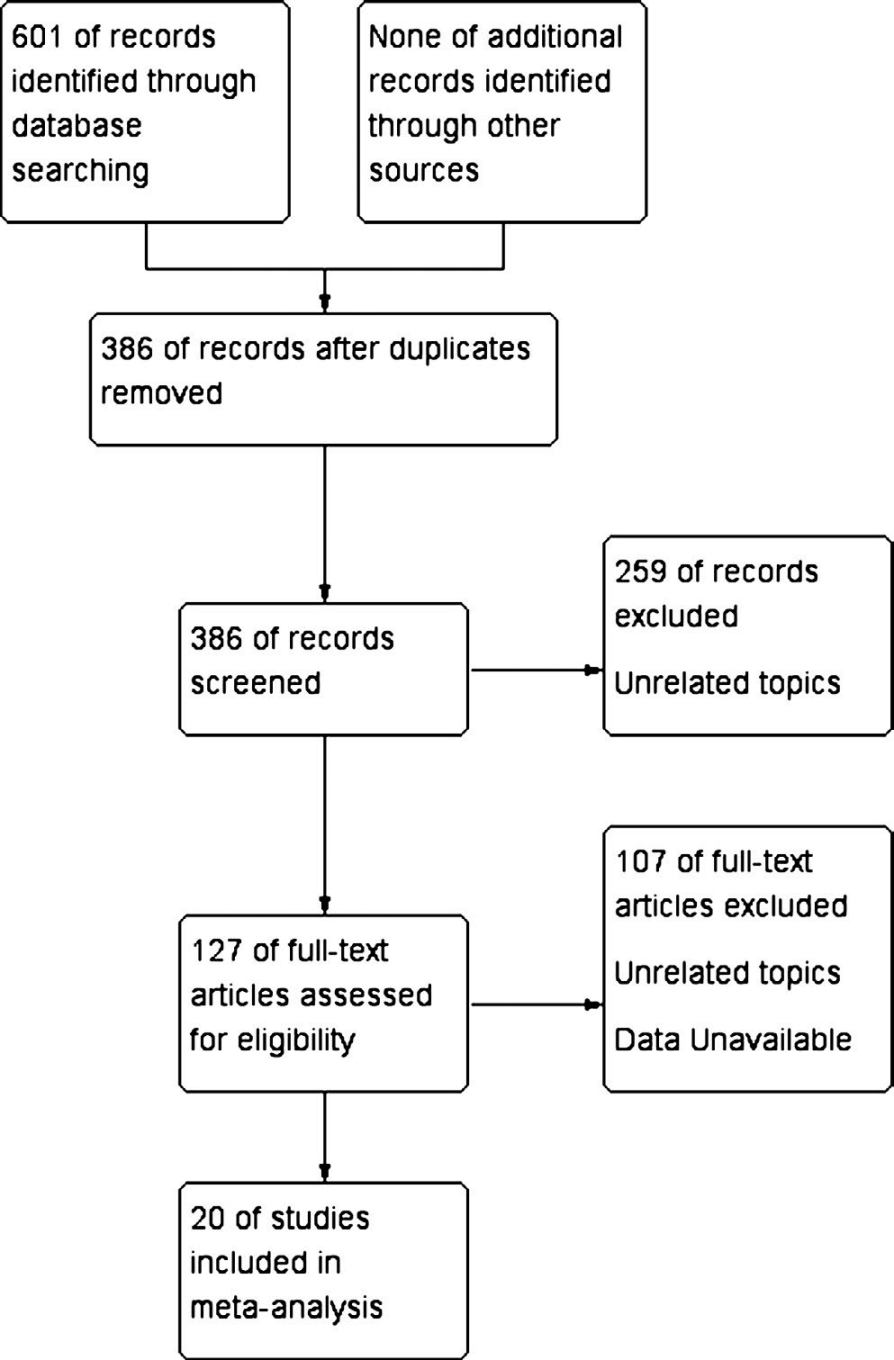
Flow chart of studies selection

A flow chart depicting the selection process is presented in Figure 1. Briefly, from the initial search, a total of 601 potentially relevant studies were identified until Feb 2020. After excluding 217 duplicated articles, the title and abstract of the remaining 386 articles were reviewed, and 259 unrelated articles were excluded. A full‐text review of the last 127 articles excluded those articles that could not obtain data and did not conform to the type of research. A total of 20 studies met the requirements of meta‐analysis.^8^, ^9^, ^10^, ^11^, ^12^, ^13^, ^14^, ^15^, ^16^, ^17^, ^18^, ^19^, ^20^, ^21^, ^22^, ^23^, ^24^, ^25^, ^26^

### 3.2 Study characteristics and quality assessment

The characteristics of the studies included are shown in Table 1, including the first author, year of publication, research design, sample size, and outcome, etc Of all, 13 studies were analyzed with complications following PN as the outcome of interest. Most studies are retrospective, only two are prospective.

**Table 1:** Characteristics of the included studies

Author	Year	Country	Journal	Study design	Patients size	Age (y)	LC	MC	HC	Outcome accessed	Surgical technique	Surgical procedure
Kutikov et al	2009	American	J Urol	Retrospective analysis	50	NA	9	19	22	Surgical strategy	Open Robotic Laparoscopic	PN RN
Hayn et al	2011	American	BJU Int	Retrospective analysis	141	60	43	91	7	Complications	Lparoscopic	PN
Canter et al	2011	American	Urology	Retrospective analysis	615	59	128	281	206	Surgical strategy	Open Minimally invasive	PN RN
Simhan et al	2011	American	Eur Urol	Retrospective analysis	390	58	109	217	64	Complications	Open Laparoscopic	PN
Rosevear et al	2012	American	BJU Int	Retrospective analysis	249	58	65	113	71	Surgical strategy	Open Laparoscopic	PN RN
Liu et al	2013	China	World J Urol	Retrospective analysis	179	57	103	74	2	Complications	Robotic Laparoscopic	PN
Kobayashi et al	2013	Japan	Urol Int	Retrospective analysis	100	64	23	65	12	Complications	Open	PN
Long et al	2013	France	BJU Int	Retrospective analysis	153	55	69	71	13	Complications	Open Laparoscopic	PN
Roushias et al	2013	Britain	Curr Urol	Retrospective analysis	128	NA	55	68	5	Complications	Open Laparoscopic	PN
Ellison et al	2013	American	Int J Urol	Retrospective analysis	290	57	135	155	NA	Complications	Robotic Laparoscopic	PN
Reddy et al	2014	Britain	Ann R Coll Surg Engl	Retrospective analysis	128	67	44	74	10	Complications	Open Laparoscopic	PN
Park et al	2014	Korea	Can Urol Assoc J	Retrospective analysis	98	54	16	48	34	Complications	Open	PN
Oh et al	2014	Korea	Korean J Urol	Retrospective analysis	206	60	58	85	63	Surgical strategy	Open Laparoscopic	PN RN
Davidiuk et al	2015	American	Urology	Prospective study	100	63	38	49	13	Complications	Robotic	PN
Benadiba et al	2015	France	Ann Surg Oncol	Retrospective analysis	52	55	16	31	5	Complications	Open Robotic Laparoscopic	PN
Shaaban et al	2015	Egypt	The Egyptian Journal of Radiology and Nuclear Medicine	Prospective study	40	NA	10	9	18	Surgical strategy	Open	PN RN
Tobert et al	2015	American	Urol Oncol	Retrospective analysis	276	61	85	115	76	Surgical strategy	NA	PN RN
Shin et al	2015	Korea	PLoS One	Retrospective analysis	1106	55	318	459	329	Surgical strategy	Open Robotic Laparoscopic	PN RN
Zhou et al	2017	China	Chin Med J (Engl)	Retrospective analysis	139	52	74	50	15	Complications	Laparoscopic	PN
Schiavina et al	2017	Italy	BJU Int	Retrospective analysis	277	60	118	139	20	Complications	Robotic	PN

Abbreviations: HC, high complexity; LC, low complexity; MC, moderate complexity; PN, partial nephrectomy; RN, radical nephrectomy.

Scores for each study are shown in Table 2. According to the Newcastle‐Ottawa Scale, we evaluated the quality of included case‐control studies and cohort studies separately. The total score was 9 stars, all studies were considered to be of medium to high quality, and no study was assessed less than 6 stars. Some studies did not report whether cohorts were matched to control confounding factors, which could lead to bias risk.

**Table 2:** Quality evaluation of included studies

Case‐control studies									
Author	Selection				Comparability	Exposure			Total
	1	2	3	4		5	6	7	
Kutikov et al	*	*		*		*	*	*	6
Rosevear et al	*	*		*	**	*	*	*	8
Long et al	*	*		*	*	*	*	*	7
Oh et al	*	*		*	*	*	*	*	7
Shin et al	*	*		*	*	*	*	*	7

Cohort studies									
Author	Selection				Comparability	Outcome			Total
	1	2	3	4		5	6	7	
Hayn et al	*	*	*		**	*	*	*	8
Canter et al	*	*	*			*	*	*	6
Simhan et al	*	*	*		*	*	*	*	7
Liu et al	*	*	*		**	*	*		7
Kobayashi et al	*	*	*			*	*	*	6
Roushias et al	*	*	*			*	*	*	6
Ellison et al	*	*	*		**	*	*		7
Reddy et al	*	*	*			*	*	*	6
Park et al	*	*	*		**	*	*	*	8
Davidiuk et al	*	*	*	*		*	*	*	7
Benadiba et al	*	*	*			*	*	*	6
Shaaban et al	*	*	*	*		*	*	*	7
Tobert et al	*	*	*			*	*	*	6
Zhou et al	*	*	*		**	*	*	*	8
Schiavina et al	*	*	*		*	*	*	*	7

### 3.3 Meta‐analysis of renal score in determining surgical strategies

**Figure 2:**
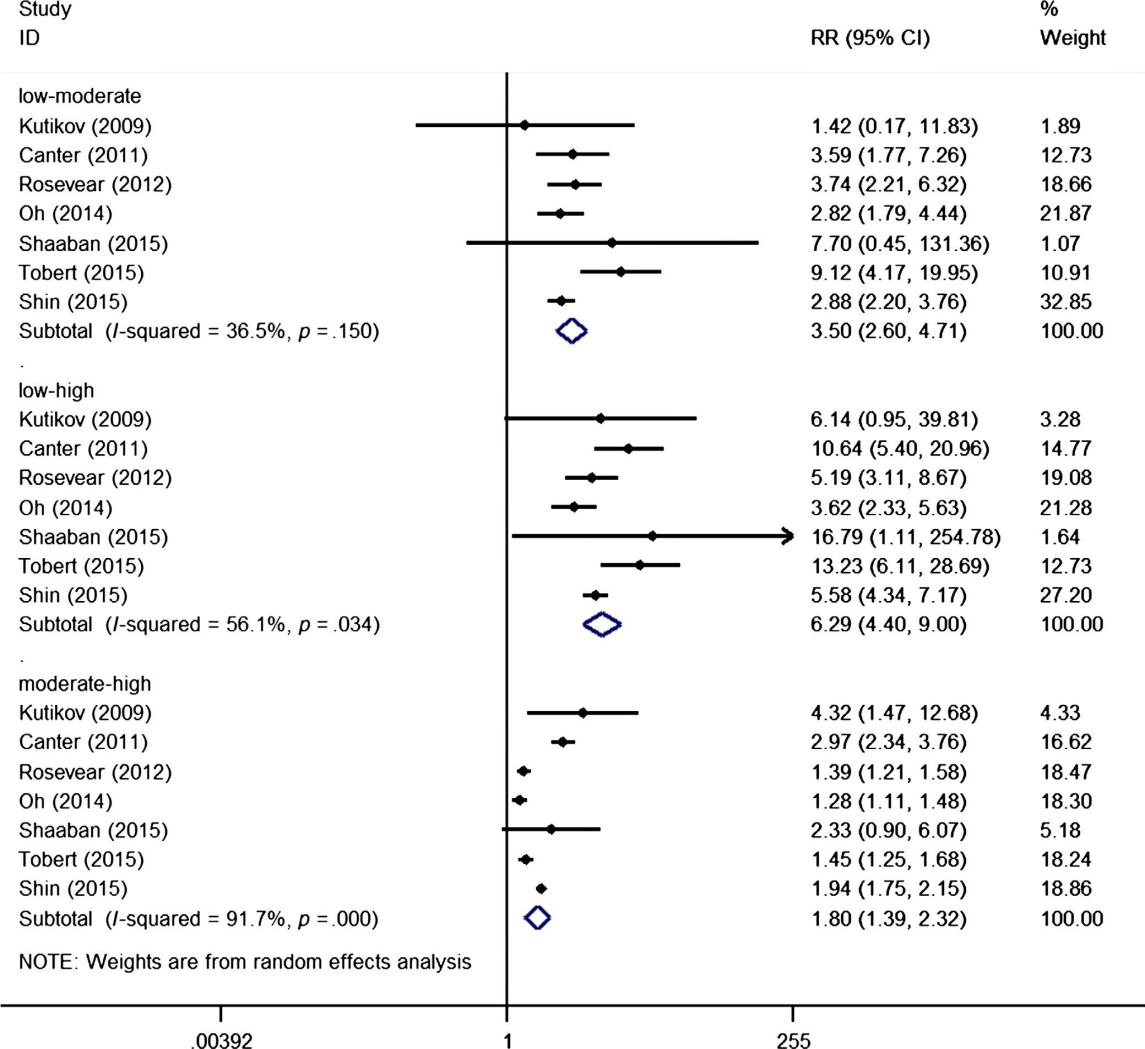
Forest plot of renal score on surgical strategies

There was significant difference between RN operation rates in each subgroup (low‐moderate: RR = 3.50, 95% Cl = 2.60 −4.71, *P* < .001; low‐high: RR = 6.29, 95% Cl = 4.40 −9.00, *P* < .001; moderate‐high: RR = 1.80, 95% Cl = 1.39 −2.32, *P* < .001). The pooled effect showed that the relatively high renal score group had a significantly higher RN operation rate than the relatively low renal score group (Figure 2). Large heterogeneity was observed in moderate‐high pair with I^2^ reaching 91.7%. Given the small number of studies, meta‐regression analysis was not performed to explore confounding factors affecting surgical strategy.

### 3.4 Meta‐analysis of renal score in predicting complications

**Figure 3:**
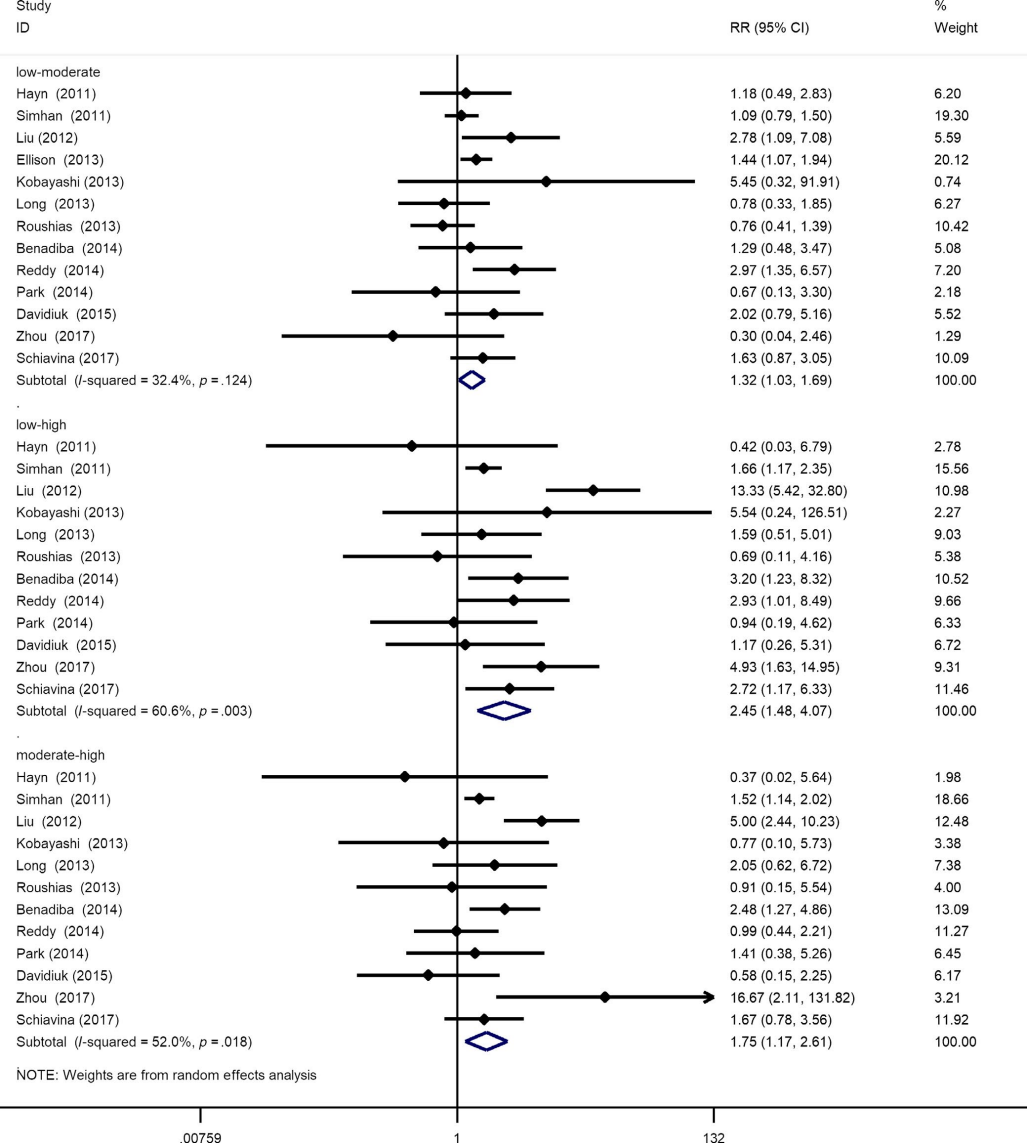
Forest plot of renal score on complications

As shown in Figure 3, the overall incidence of complications in high renal score group was significantly higher than that in low renal score group (low‐moderate: RR = 1.32, 95% Cl = 1.03 −1.69, *P* = .026; low‐high: RR = 2.45, 95% Cl = 1.48 −4.07, *P* = .001; moderate‐high: RR = 1.75, 95% Cl = 1.17 −2.61, *P* = .007). Meta‐regression analysis was conducted with surgical techniques (minimal invasive vs open) as covariate, which is considered to be a possible factor for postoperative complications. The results showed that the effect of renal score on complications is not disturbed by surgical techniques (Table 3).

**Table 3:** Meta regression analysis of surgical techniques in each complications pair

logRR	Coef	SE	*t*	*P *> |*t*|	[95% CI]	
Low‐moderate						
MIS	0.3064115	0.2117803	1.45	0.179	−0.1654643	0.7782873
Open	−0.000592	0.870256	−0.00	0.999	−1.939643	1.938459
_cons	0.1059483	0.1522118	0.70	0.502	−0.2332008	0.4450973
Low‐high						
MIS	0.6800205	0.5141472	1.32	0.219	−0.4830612	1.843102
Open	−0.2796674	0.9499079	−0.29	0.775	−2.428508	1.869173
_cons	0.6566342	0.34114	1.92	0.086	−0.115078	1.428346
Moderate‐high.						
MIS	0.3836185	0.4974288	0.77	0.460	−0.7416435	1.508881
Open	−0.3121784	0.7948689	−0.39	0.704	−2.110297	1.48594
_cons	0.4488784	0.3142575	1.43	0.187	−0.2620215	1.159778

Abbreviation: MIS, minimally invasive surgery.

### 3.5 Sensitivity analysis and publication bias

To evaluate the stability to the conclusion of this meta‐analysis, we conducted a sensitivity analysis by dropping each study sequentially, and the sensitivity analysis indicated that the results of the meta‐analysis were robust. We tested the publication bias of each pairs for two outcomes using the egger test of Stata software. The results showed that *P*  > .05 in all pairs, and there was no significant publication bias.

## 4 DISCUSSION

Researches supporting similar postoperative outcomes of PN and RN have contributed to the popularity of PN, which can better preserve renal function.^27^ However, studies have shown that there is a higher incidence of complications after PN, including hemorrhagic complications and urinary leakage. Other studies have shown that PN reduces the risk of chronic kidney disease compared with RN.^28^ Nowadays, renal score based on the development of imaging technology can objectively evaluate the surgical difficulty of renal cell carcinoma to help clinicians choose appropriate surgical strategies.

In this meta‐analysis, we found that renal score was significantly correlated with the surgical strategies in renal cell carcinoma patients. The higher the renal score, the more likely people were to undergo RN surgery. The results of the three subgroups were consistent. The risk of RN surgery in the high group was more than five times higher than that in the low group (RR = 6.29, 95% Cl = 4.40 −9.00, *P* < .001). This is because the higher the renal score, the more difficult the PN is, and the worse the prognosis of PN is. Although renal score was significantly correlated with surgical strategies, not all components were in this way. Rosevear et al reported that only three components of R, N, and L are significantly related to the choice of surgical strategies.^11^ However, Shin et al reported that there is no significant correlation between L (location relative to the polar line) and surgical strategies, and the H (+) proportion among the partial nephrectomy cases increased.^24^ In order to clarify the correlation between the components of renal score and surgical strategies, a more comprehensive multivariate logistic regression analysis is needed. Oh et al also noted that the choice of laparoscopic vs open radical nephrectomy depended upon the R and L scores, while the choice of laparoscopic vs open partial nephrectomy depended upon the E score.^19^ It's also controversial. The value of renal score in decision‐making for open or minimally invasive surgery is not clear. With the development of minimally invasive technology, the answer to this question may be more ambiguous.

The study also found that there was a relationship between the renal score and the incidence of complications. The risk of complications in the high‐complex tumor group was almost 2.5 times that of the low‐complex group (RR = 2.45, 95% Cl = 1.48 −4.07, *P* = .001). Tanagho et al reported that tumor diameter is a predictor of complications.^29^ Schiavina et al reported that clinical tumor size was significantly correlated to grade 3‐4 postoperative complications.^26^ Patients in the high complexity group have larger tumor size than those in the low complexity group. In our study, we also found that renal score was related to the openness of the collection system and estimated blood loss (EBL). The higher the tumor complexity, the larger EBL, and the closer the tumor is to the urinary collection system, which has been reported as a risk factor for postoperative complications.^30^ Tomaszewski et al reported that urinary collection system opening is a risk factor for urinary leakage.^31^ Warm ischemia time is also prolonged in high complex tumors, suggesting that it may also be a predictor of complications. In addition, the length of hospital stay is also higher in the high‐complexity tumor group, which may increase the risk of nosocomial infections or pneumonia and pressure ulcers in patients. Meta‐regression analysis showed no statistical significance of surgical technique on the predictive effect of renal score on complications. Our results can be extended to the general population undergoing PN surgery regardless open or laparoscopic surgery. In fact, the relationship between surgical techniques and complications remains controversial. Recently, a prospective multicenter study reported that minimally invasive surgery had lower rate of Clavien–Dindo ≥ 2 complications than that of open surgery.^32^ In contrast, Ng et al reported in a narrative review that laparoscopic partial nephrectomy share the equivalent incidence of complications as open partial nephrectomy.^33^ Patients’ age and surgeon's experience have also been reported as possible predictors of complications,^34^ but in our meta‐analysis, the baseline data of patients did not match, and surgeon's experience could not be quantified, which could be responsible for heterogeneity.

Renal score is a useful systematic tool for assessment of the anatomical features of the renal tumors, consisting of (R)adius (tumor size as maximal diameter), (E)xophytic/endophytic properties of the tumor, (N)earness of tumor deepest portion to the collecting system or sinus, (A)nterior (a)/posterior (p) descriptor, and the (L)ocation relative to the polar line.^7^ It is easily reproducible and applicable by different radiologists of different durations of experience.^35^ Except for renal scoring system, PADUA and centrality index (C index) have been used to evaluate the anatomical characteristics of renal cell carcinoma.^36^, ^37^ PADUA scoring system is very similar to renal score. It contains seven tumor parameters, namely: (a) anterior or posterior face, (b) longitudinal location, (c) rim location; (d) relationships with sinus; (e) relationships with the collecting system; (f) percentage of tumor deepening into the kidney; and (g) maximal diameter in centimeters. Unlike the renal score, the PADUA score does not use polar lines to describe the tumor's coronal location, but uses a more recognizable renal sinus line. In addition, the relationship between tumor and renal sinus or collective system is no longer described by distance. C index is different from the former two, it is calculated based on the tumor radius and the distance from the tumor center to the kidney center. It is generally believed that the larger the value of C index, the farther the tumor is from the renal center. It has been shown to be effective in assessing the location of renal tumors and in predicting the incidence of complications. All three scoring systems demonstrated reliability among observers and represent novel methods of quantitatively describing renal tumors.^38^ Comparative studies of the three scoring systems on perioperative outcome in PN have also been published widely, predictive role of them can be further verified by network meta‐analysis. Kopp et al published a meta‐analysis of renal score in predicting recurrence and metastasis of tumors after surgery in 2013.^39^ The current meta‐analysis explored the relationship between renal score and surgical strategies and complications. It is confirmed that renal score can be used as an effective evaluation tool to help clinicians make surgical decisions and predict complications in PN.

Limitations exist in the current study. Great heterogeneity was found in moderate‐high pair with surgical strategy as outcome, which may be attributed to short 95% CI. But statistically, this also shows the validity of the conclusion that the renal score is significantly related to the surgical strategy. In addition, it is impossible to account for the surgical techniques. We cannot discuss laparoscopic surgery and open surgery separately due to limitation of included studies. However, we performed a meta‐regression analysis to confirm that surgical technique has no statistical impact on the effect of renal score. Moreover, most of the included studies are retrospective studies with nature of bias, which is the biggest limitation of this meta‐analysis. Including more high‐quality prospective study can be a powerful evidence for renal score as an effective assessment and prediction tool, which provides a direction for future work.

## 5 CONCLUSIONS

Renal score is an effective, objective, and reproducible assessment tool, which is of great value for decision‐making of surgical strategies and predicting complications in partial nephrectomy. High‐quality prospective study can contribute to proving the predictive value of renal score.

## CONFLICT OF INTEREST

There are no known conflicts of interest associated with this publication.

## Data Availability

The data that support the findings of this study are available from the corresponding author upon a reasonable request.
